# The impact of home-based HIV counseling and testing on care-seeking and incidence of common infectious disease syndromes in rural western Kenya

**DOI:** 10.1186/1471-2334-14-376

**Published:** 2014-07-08

**Authors:** Godfrey Bigogo, Manase Amolloh, Kayla F Laserson, Allan Audi, Barrack Aura, Warren Dalal, Marta Ackers, Deron Burton, Robert F Breiman, Daniel R Feikin

**Affiliations:** 1 Center for Global Health Research, Kenya Medical Research Institute, P.O. Box 1578, 40100 Kisumu, Kenya; 2 International Emerging Infections Program – Kenya, Centers for Disease Control & Prevention, Nairobi, Kenya; 3 Center for Global Health, Centers for Disease Control & Prevention, Atlanta, GA, USA; 4 Division of Global HIV and AIDS, Centers for Disease Control & Prevention, Atlanta, GA, USA

**Keywords:** Home based HIV counseling and testing, Infectious disease incidence, Healthcare seeking

## Abstract

**Background:**

In much of Africa, most individuals living with HIV do not know their status. Home-based counseling and testing (HBCT) leads to more HIV-infected people learning their HIV status. However, there is little data on whether knowing one’s HIV-positive status necessarily leads to uptake of HIV care, which could in turn, lead to a reduction in the prevalence of common infectious disease syndromes.

**Methods:**

In 2008, Kenya Medical Research Institute (KEMRI) in collaboration with the Centers for Disease Control and Prevention (CDC) offered HBCT to individuals (aged ≥13 years) under active surveillance for infectious disease syndromes in Lwak in rural western Kenya. HIV test results were linked to morbidity and healthcare-seeking data collected by field workers through bi-weekly home visits. We analyzed changes in healthcare seeking behaviors using proportions, and incidence (expressed as episodes per person-year) of acute respiratory illness (ARI), severe acute respiratory illness (SARI), acute febrile illness (AFI) and diarrhea among first-time HIV testers in the year before and after HBCT, stratified by their test result and if HIV-positive, whether they sought care at HIV Patient Support Centers (PSCs).

**Results:**

Of 9,613 individuals offered HBCT, 6,366 (66%) were first-time testers, 698 (11%) of whom were HIV-infected. One year after HBCT, 50% of HIV-infected persons had enrolled at PSCs – 92% of whom had started cotrimoxazole and 37% of those eligible for antiretroviral treatment had initiated therapy. Among HIV-infected persons enrolled in PSCs, AFI and diarrhea incidence decreased in the year after HBCT (rate ratio [RR] 0.84; 95% confidence interval [CI] 0.77 – 0.91 and RR 0.84, 95% CI 0.73 – 0.98, respectively). Among HIV-infected persons not attending PSCs and among HIV-uninfected persons, decreases in incidence were significantly lower. While decreases also occurred in rates of respiratory illnesses among HIV-positive persons in care, there were similar decreases in the other two groups.

**Conclusions:**

Large scale HBCT enabled a large number of newly diagnosed HIV-infected persons to know their HIV status, leading to a change in care seeking behavior and ultimately a decrease in incidence of common infectious disease syndromes through appropriate treatment and care.

## Background

After three decades of the HIV epidemic in sub-Saharan Africa, in many parts of the continent many people still do not know their HIV status [1-5]. This is due to multiple factors including low perception of risk, distance to HIV testing centers, costs incurred in getting tested, and ongoing stigma of being HIV-infected [6,7]. Two national AIDS indicator surveys have been done in Kenya. In the first survey in 2007, only 34% of adults nationwide had been tested before, and among those found to be HIV-infected, only 16% correctly reported their HIV status. These proportions increased to 72% and 47% respectively in the second survey in 2012 [1,2,4]. Without knowledge of their HIV status HIV-infected persons do not access HIV care and treatment services, including cotrimoxazole prophylaxis for opportunistic infections and antiretroviral therapy (ART), nor are they likely to receive adequate treatment for acute illness episodes [8-11].

Because the majority of HIV-infected persons not in care are those that do not know their HIV status, mass initiatives to test more persons for HIV are needed [12]. One strategy for increasing the number of individuals who know their HIV status is home-based HIV counseling and testing (HBCT) [4,13,14]. HBCT can overcome barriers of cost, time-expenditure and stigma that prevent many persons in rural Africa from seeking voluntary counseling and testing (VCT) at fixed facilities [13-15]. While it has been shown that HBCT can increase HIV testing and the number of people who know their HIV status, it is not clear whether knowledge of HIV status influences care seeking which in turn impacts incidence of common infectious illnesses such as acute respiratory illness, febrile illness and diarrhea whose burden is high in several parts of Africa [16]. We hypothesize that as more people know their HIV status, enroll into HIV care and treatment, and receive highly-active antiretroviral treatment and cotrimoxazole prophylaxis that the incidence of infectious disease morbidity will decrease in the community. We further hypothesize that as people newly learn their HIV-status they will be more likely to seek care for common infectious disease syndromes, either because their access to care has improved or their health awareness and concern has increased due to learning their HIV status. We describe the impact of a large-scale HBCT program among newly diagnosed HIV-infected residents in the year after HBCT in a high HIV prevalence population under ongoing surveillance for common infectious disease syndromes in rural western Kenya.

## Methods

### Study site and surveillance population

**Figure 1:**
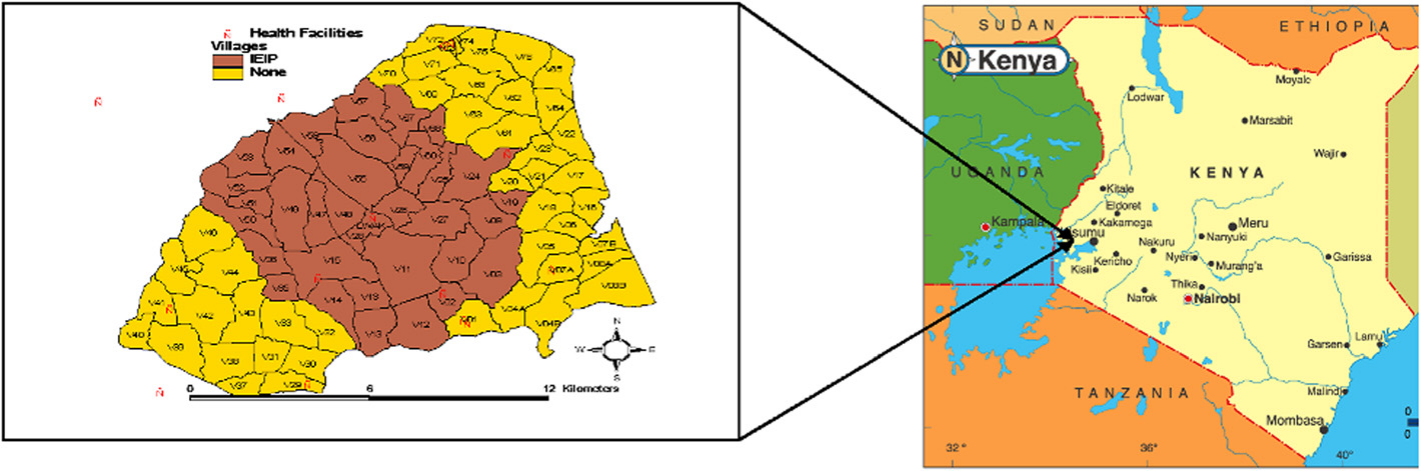
KEMRI/CDC’s Population based disease surveillance site in rural western Kenya.

Since 2005, the Lwak area has been the site of population-based infectious disease surveillance (PBIDS) run by the Kenya Medical Research Institute (KEMRI) in collaboration with the U.S. Centers for Disease Control and Prevention (CDC), (Figure 1). The PBIDS catchment area has an average population of 25,000 people living in 33 villages that are within a 5 kilometer radius of Lwak Hospital, the project’s designated referral health facility. The Lwak area is culturally homogeneous (95% Luo ethnicity), and poor, with the primary economic livelihood being subsistence farming and fishing [17,18]. Houses are predominantly clustered into compounds comprised of houses for the male head of household, his wives, and sons. Compounds are dispersed and lie adjacent to the households’ agricultural fields. There are a few clay or soil roads in the area and one tarmac road, completed in 2006. The most common mode of transportation is by walking or bicycle taxi.

According to the most recent national HIV/AIDS survey in 2012, Nyanza Province, where Lwak PBIDS is located, has the highest HIV prevalence in Kenya – 15% for the adult population [1,4]. The first HIV/AIDS survey in 2007 showed that only 34% of adults aged 15 years and above in the province had ever been tested before for HIV. However, the 2012 survey showed a marked increase to 72%. The PBIDS area also has one of the highest rates of malaria in Africa. In fact, it is holoendemic, − almost everyone in the population is infected with malaria [16,19].

### Household surveillance

PBIDS household surveillance methods have been described previously [16,20]. In brief, community interviewers visit enrolled households every two weeks. The catchment population includes people living within a 5 km radius of Lwak hospital, the project’s referral facility. Participants are asked standardized questions, in the local language, about recent illnesses and care seeking. For certain key symptoms – cough, fever and diarrhea – the exact days of occurrence are recorded. For older children (over 12 years old) and adults, interviews of that person are done. If not at home or unable to answer questions (i.e. children ≤ 12 years old and cognitively impaired persons), a proxy who is knowledgeable about the participant’s health completes the interview. Abbreviated physical exams are carried out on ill persons present during the visit, including axillary temperature and observation for signs of dehydration.

### Home-based counseling and testing

In early 2008 HBCT services were initiated and offered to 12,149 PBIDS residents free of charge in their homes; 9,613 (79%) accepted and were HIV-tested [15]. To participate in HBCT, one needed to be a resident in the PBIDS area and also provide consent for participation. A team of trained non-resident HIV counselors provided HIV testing and counseling at home to residents ≥13 years of age who gave consent [13,15]. Rapid HIV testing was done according to the National AIDS/STD Control Program (NASCOP) algorithm using two parallel HIV rapid tests (Determine™, Abbott Laboratories, USA) and Bioline™ (Standard Diagnostics, Kyonggi-do, Korea) with a tie-breaker test for discordant results (Uni-gold™, Trinity Biotech, Ireland). For individuals newly diagnosed with HIV counselors collected additional finger-prick blood samples for CD4 testing [21]. These blood samples were tested at the KEMRI/CDC laboratories in Kisumu, and results made available at Lwak Hospital within five days. One month following HBCT, the same counselors made follow-up visits to newly diagnosed HIV-infected persons to offer further counseling and deliver CD4 results. At the follow-up visits, we collected data on whether the clients had visited an HIV Patient Support Center (PSC) and whether they had started any HIV treatment since HBCT. Counselors also encouraged those that had not enrolled in HIV care to do so at any of the four nearby clinics within the PBIDS area where free HIV care and treatment services were provided.

### Clinical care

Since 2005, PBIDS residents have received free medical care at Lwak Hospital for all acute, potentially infectious disease conditions by KEMRI/CDC-trained clinical officers (equivalent to physician assistants) and nurses.

In 2007 Lwak Hospital, through support from the President’s Emergency Plan for HIV/AIDS Relief (PEPFAR), began providing free HIV care and treatment services. At the start in 2007, the clinic was only operational once a week on Mondays, and later increasing operations to five days a week in 2008 with an expanded team of nurses, clinical officers and counselors. HIV-infected clients could also continue to access free clinical care for illnesses through the PBIDS staff, as both the PBIDS clinic and the PSC were located at Lwak Hospital. PBIDS residents who chose to access services at any of the three other health facilities could receive free HIV care and treatment services, but unlike at Lwak would need to pay facility fees for acute illness episodes.

### Data collection and analysis

HIV testing and home follow-up visit data were collected on paper forms (TeleForm®, Cardiff™, Vista, CA), and later scanned and stored in Microsoft Access. PBIDS data were collected using personal digital assistants (PDAs) [20,22]. Medical chart data on those PBIDS residents enrolling in HIV care at Lwak Hospital were linked to other data using unique ID numbers. Data from the other three health facilities were abstracted using a standardized form and linked back to individuals’ HBCT data through the use of unique ID numbers when available, and when not available, through a combination of names, gender, age/date of birth and place of residence.

Our analysis was restricted to first-time testers who also had data from household morbidity surveillance available; therefore, the included sample was slightly different from that of previously reported results from HBCT [15]. We focus the analysis on first-time testers because they provide the clearest indication of how learning one’s HIV status affects health-seeking and disease incidence.

We calculated the pre and post-HBCT incidence of four syndromes namely acute respiratory illness (ARI), severe acute respiratory tract illness (SARI), acute febrile illness (AFI) and diarrhea using data from the household visits. We defined ARI as cough or difficult breathing. The definition of SARI included documented fever with either cough or difficult breathing or chest pain [23]. AFI was defined as the presence of subjective or documented fever. Diarrheal illness was defined as an illness in which at least three looser than normal stools were reported during a 24-hour period. Each of these syndromes was measured at a binary level as being either present or absent. Using the biweekly household visit data, we calculated incidence, as the number of illness episodes per person-year in pre-HBCT and post-HBCT periods. We used three symptom free days as the minimum interval to distinguish different episodes of diarrhea and seven symptom free days to distinguish episodes of respiratory and febrile illnesses [16]. The pre-HBCT period included episodes recorded in the year before the HIV test date for each individual, while the post-HBCT captured illnesses in the year following HIV testing. PBIDS interviewers were blinded to respondents HIV status. Because the annual HIV incidence is estimated as 1.5% in the area (KEMRI/CDC unpublished data), we assumed an individual’s HIV status at HBCT would likely be the same in both pre- and post-HBCT periods. The person-time contribution was computed for each individual both pre- and post-HBCT, which were summed to give the denominator for incidence calculations. Using household data, we compared pre- and post-HBCT proportions of each syndrome among persons seeking care. We did not evaluate incidence of the syndromes based on clinic visitation. This was because if health-seeking to the clinic changed after HBCT, as we speculated, then rates of disease syndromes would reflect changes in health-seeking rather than true changes in disease incidence. To measure changes in severe disease, we used rates of hospitalization, which we felt were less subject to temporal changes in health-care seeking since standardized clinical criteria for hospitalization irrespective of HIV status were used. We compared pre-post HBCT incidence among three groups of individuals – newly HIV-infected and enrolled in HIV care, newly HIV-infected and not enrolled in HIV care, and HIV-uninfected persons. The first two groups were compared to reveal whether any changes in morbidity could be ascribed to enrolling into HIV care programs, rather than to changes in reporting or perception of illness that occurred by just testing positive in HBCT. The HIV-negative group was included to control for secular changes in the health-care seeking and disease incidence in the area that were unrelated to HBCT.

For each syndrome in each period, we calculated incidences, adjusted for age and sex. We controlled for clustering of symptoms at the household level using generalized estimating equations (GEE) [16]. Rates and rate ratios were calculated using Poisson regression (PROC GENMOD, SAS version 9.1, SAS Institute, Cary, North Carolina, USA.

### Ethical review

The protocol and consent forms were reviewed and approved by the Ethical Review Boards of KEMRI (SSC 932) and the Institutional Review Board of CDC (IRB 4566). Signed participant consent was obtained for linking of HIV data to individuals’ morbidity and demographic data.

## Results

Of 9,613 individuals aged ≥13 years who received HIV testing between January 2008 and February 2009, 6,366 (66%) participants were tested for their first time – 57% being female, (Table 1). Overall HIV prevalence among first-time testers ≥13 years was 10.9%; 8.6% in males versus 12.7% in females and peaked in people in 35 – 49 year age group.

**Table 1:** Characteristics of HBCT participants receiving first-time HIV testing in western Kenya, 2008 – 2010

**Age**	**Males n/N (% HIV-infected)**	**Females n/N (% HIV-infected)**	**Overall n/N (% HIV-infected)**
13-17	13/917 (1.4)	37/821 (4.5)	50/1738 (2.9)
18-34	93/888 (10.5)	215/975 (22.1)	308/1863 (16.5)
35-49	79/340 (23.2)	132/612 (21.6)	211/952 (22.2)
≥50	51/593 (8.6)	78/1220 (6.4)	129/1813 (7.1)
Total	236/2738 (8.6)	462/3628 (12.7)	698/6366 (10.9)

N = No. of people tested for their first time ever; n = no. with HIV.

**Figure 2:**
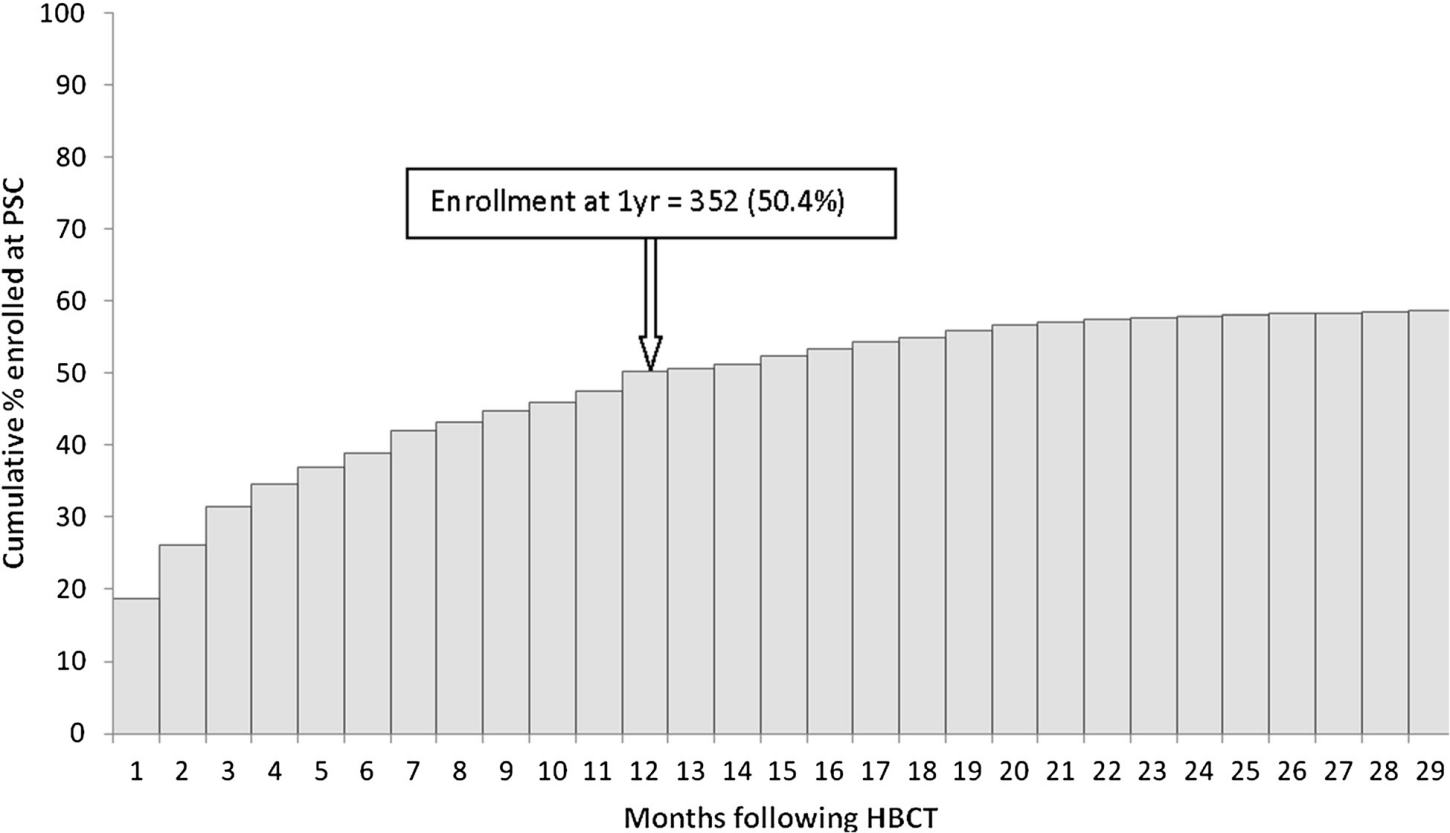
Cumulative enrollment for HIV care at various clinics following HBCT in HIV-infected first-time lesters, western Kenya, 2008-2010.

**Figure 3:**
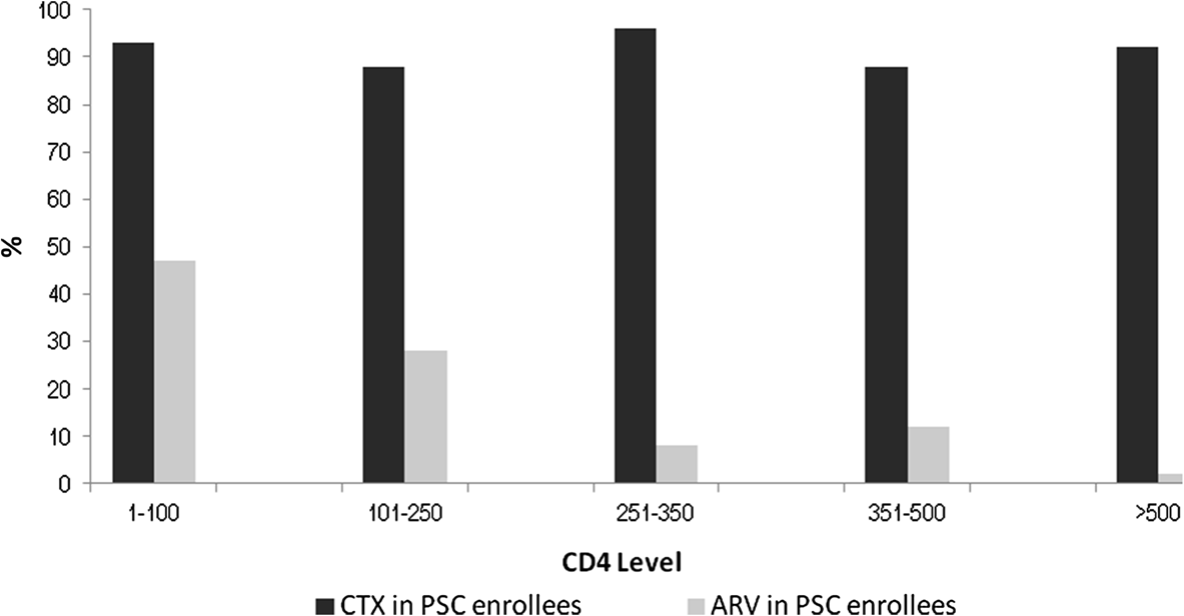
**CTX/ARV use by CD4 level for HIV-infected first-time testers aged ≥13 years enrolled for HIV care, western Kenya, 2008-2010.** Guidelines in 2008 in Kenya recommend all HIV-infected persons with CD4 < 250 be started on ARVs.

Among the 698 HIV-infected first-time testers, 219 (31%) had enrolled in HIV care by 3 months after HBCT, 50% had enrolled at one year and 58% by 24 months (Figure 2). Over half (56%) who enrolled in care enrolled at Lwak Hospital. There were no age or sex differences in the proportions of HIV-infected people enrolled at PSCs versus those HIV-infected but not enrolled (data not shown). CD4 counts were available from 635 (91%) of the first-time testers. No significant differences were observed in the median CD4 counts for those who enrolled for HIV care versus those who did not (402 cells/mm^3^, inter-quartile range 240 – 616 cells/mm^3^ versus 485 cells/mm^3^, inter-quartile range 347 – 583 cells/mm^3^). Of those enrolled at the PSCs according to national guidelines at the time, 92% were initiated on cotrimoxazole prophylaxis and 37% of people with CD4 counts ≤250 cells/mm3 started taking antiretroviral drugs (ARVs) (Figure 3).

In all syndromes examined, more disease was reported among HIV-infected persons than among uninfected persons in the pre-HBCT period – 10% more for ARI, 25% more for AFI, 86% more diarrhea and 274% more for SARI (Table 2). Overall, among HIV-infected persons 18.4% of household visits had an illness episode reported in the year pre-HBCT and 17.2% in the year post-HBCT (Table 3). In comparison, among HIV uninfected persons 15.6% of household visits had an illness episodes reported in the year pre-HBCT and 13.5% in the year post-HBCT. ARI and AFI were the most common syndromes in both periods for both HIV-infected and uninfected persons. Several notable trends were found in health-seeking for common illnesses (Table 3). First, the percentage of episodes resulting in a clinic visit was higher for all syndromes among HIV-infected than uninfected persons, both before and after HBCT. Second, the percentage of episodes resulting in a clinic visit increased after HBCT for all four syndromes among HIV-infected persons, although only significantly for ARI (4.8% increase, [95% CI 2.3% – 7.3%]) and AFI (9.7% increase, [95% CI 6.6% – 12.8%]). Health-seeking among HIV-infected persons increased mostly at the designated PBIDS facility, Lwak Hospital. Lastly, health-seeking increased more from pre- to post-HBCT periods among HIV-infected persons than uninfected persons for episodes of ARI (5.2% greater increase, [95% CI 0.1% – 10.3%]) and AFI (7.0% greater increase, [95% CI 0.6% – 13.4%).

**Table 2:** Comparing disease rates as episodes per person-year of observation (pyo) in pre-HBCT period among all first-time testers with and without HIV infection based on household morbidity data, western Kenya, 2007-2009

**Syndrome**	**HIV-infected episodes/pyo**	**HIV-uninfected episodes/pyo**	**Rate ratio (95% CI) (HIV infected/uninfected)**
ARI	4.31	3.92	1.10 (1.06 – 1.14)
SARI	0.06	0.02	3.74 (1.83 – 7.66)
AFI	4.21	3.35	1.25 (1.13 – 1.40)
Diarrhea	1.50	0.81	1.86 (1.57 – 2.19)

ARI = Acute respiratory illness. SARI = Severe Acute Respiratory Illness. AFI = Acute febrile illness.

**Table 3:** Use of health care facilities by syndrome pre- and post-HBCT for first-time HIV testers, stratified by HIV infection status, based on household morbidity surveillance data, western Kenya 2007 – 2010

	**HIV-infected persons, N = 698**			**HIV-uninfected persons, N = 5,668**			
	**Pre-HBCT**	**Post-HBCT**	**Diff. in % (95% CI)**	**Pre-HBCT**	**Post-HBCT**	**Diff. in % (95% CI)**	Diff. of diffs*. (95% CI)
No. of household visits	15,421	16,757		128,837	138,801		
ARI Episodes	2438	2507		18385	17167		
n (%) to any clinic	563 (23.1)	701 (27.9)	4.8 (2.3 – 7.3)	2754 (14.9)	2491 (14.5)	−0.4 (−1.1 – 0.3)	5.2 (0.1 – 10.3)
n (%) to Lwak	290 (11.9)	453 (18.1)	6.2 (4.2 – 8.2)	1326 (7.2)	1367 (7.9)	0.7 (0.1 – 1.3)	5.5 (1.4 – 9.6)
n (%) to other clinics	273 (11.2)	248 (9.9)	−1.3 (−3.1 – 0.5)	1428 (7.8)	1124 (6.5)	−1.3 (−1.8 – -0.8)	0.0 (−3.7 – 3.7)
SARI Episodes	18	26		37	32		
n (%) to any clinic	5 (27.8)	11 (42.3)	14.5 (−57.6 – 28.6)	9 (24.3)	8 (25.0)	0.7 (−22.6 – 24.0)	13.8 (−82.2 – 109.8)
n (%) to Lwak	2 (11.1)	8 (30.8)	19.7 (−7.9 – 47.3)	3 (8.1)	4 (12.5)	4.4 (−12.9 – 21.8)	15.3 (−48.6 – 79.2)
n (%) to other clinics	3 (16.7)	3 (11.5)	−5.2 (−31.1 – 20.7)	6 (16.2)	4 (12.5)	−3.7 (−23.1 – 15.7)	8.9 (−54.5 – 72.3)
AFI Episodes	1746	1956		11224	13275		
n (%) to any clinic	540 (30.9)	794 (40.6)	9.7 (6.6-12.8)	2567 (22.9)	3393 (25.6)	2.7(1.6 – 3.8)	7.0 (0.6 – 13.4)
n (%) to Lwak	255 (14.6)	526 (26.9)	12.3 (9.7 – 14.9)	1175 (10.5)	1771 (13.3)	2.8 (2.0 – 3.6)	9.5 (4.2 – 14.8)
n (%) to other clinics)	285 (16.3)	268 (13.7)	−2.6 (−5.0 – -0.2)	1392 (12.4)	1622 (12.2)	−0.2(−1.0 – 0.6)	2.4 (−2.6 – 7.4)
Diarrhea Episodes	623	620		2694	2588		
n (%) to any clinic	205 (32.9)	211 (34.0)	1.1 (−4.3 – 6.5)	494 (18.3)	469 (18.1)	−0.2 (−2.3 – 1.9)	1.3 (−10.1 – 12.7)
n (%) to Lwak	116 (18.6)	146 (23.5)	4.9 (0.2 – 9.6)	249 (9.2)	263 (10.2)	1.0 (−0.6 – 2.6)	3.9 (−5.8 – 13.6)
n (%) to other clinics)	89 (14.3)	65 (10.5)	−3.8 (−7.6 – 0.0)	245 (9.1)	206 (8.0)	−1.1 (−2.7– 0.5)	2.7 (−5.4 – 10.8)

*Difference of differences is the difference between the percentage of persons with illness seeking care pre- and post-HBCT with HIV infection minus the percentage of persons with illness seeking care pre- and post-HBCT without HIV infection.

ARI = Acute respiratory illness. SARI = Severe Acute Respiratory Illness. AFI = Acute febrile illness.

There was a significant reduction in rates of AFI (16% decrease, [RR 0.84, 95% CI 0.77 – 0.91]) and diarrhea (16% decrease, [RR 0.84, 95% CI 0.73 – 0.98]) among HIV-infected individuals enrolled in a PSC in the post-HBCT period compared to the pre-HBCT period (Table 4). No difference was observed between the pre- and post-HBCT periods in rates for any syndrome in the HIV-infected group not enrolled in a PSC. In the HIV-uninfected group, there was a significant 9% reduction in ARI rate (RR 0.91, 95% CI 0.87 – 0.95), a 54% reduction in SARI (RR 0.46, 95% CI 0.23 – 0.93), an 8% reduction in the diarrhea rate (RR 0.92 95% CI 0.85 – 0.99), and an 8% increase in the AFI rate (RR 1.08, 95% CI 1.03 – 1.14), between pre- and post-HBTC periods. There was a greater reduction in rates of AFI and diarrhea among HIV-infected persons enrolled in a PSC than among either HIV-infected persons not enrolled in a PSC or HIV-uninfected persons. No significant differences were found in pre-to-post-HBCT changes in the rates of ARI or SARI between the three groups.

**Table 4:** Comparing disease incidence and hospitalization rates, (as episodes/person-year of observation (pyo)) for first-time testers in the years before and after HBCT in, I) HIV-infected individuals enrolled for HIV care, II) HIV-infected individuals not in HIV care, III) HIV-uninfected individuals, western Kenya, 2007 – 2010

	**I**			**II**			**III**				
	**HIV-infected and enrolled for HIV care (n = 351)**			**HIV-infected and not enrolled for HIV care (n = 347)**			**HIV-uninfected group (n = 5668)**				
	**Episodes/pyo**			**Episodes/pyo**			**Episodes/pyo**			**Ratio of rate ratio**	
										**(95% CI)**	
**Syndrome**	**Pre-HBCT**	**Post-HBCT**	**Rate ratio (95% CI)**	**Pre-HBCT**	**Post-HBCT**	**Rate ratio (95% CI)**	**Pre-HBCT**	**Post-HBCT ratio**	**Rate (95% CI)**	**I vs II**	**I vs III**
ARI	4.42	4.30	0.97 (0.89 – 1.06)	4.30	3.72	0.86 (0.70 – 1.06)	3.92	3.57	0.91 (0.87 – 0.95)	1.13 (0.83 – 1.53)	1.07 (0.79 –1.43)
SARI	0.06	0.03	0.46 (0.14 – 1.52)	0.05	0.02	0.32 (0.03 – 3.09)	0.02	0.01	0.46 (0.23 – 0.93)	1.44 (0.90 – 2.33)	1.00 (0.66 –1.54)
AFI	4.97	4.18	0.84 (0.77 – 0.91)	3.51	3.58	1.02 (0.82 – 1.27)	3.35	3.62	1.08 (1.03 – 1.14)	0.82 (0.74 – 0.90)	0.77 (0.70 –0.85)
Diarrhea	1.38	1.16	0.84 (0.73 – 0.98)	0.95	0.95	1.00 (0.67 – 1.51)	0.81	0.74	0.92 (0.85 – 0.99)	0.84 (0.76 – 0.92)	0.91 (0.83 – 1.00)
Hospitalization from any cause	0.07	0.19	2.57 (1.59 – 4.16)	0.08	0.15	1.74 (0.76 – 3.98)	0.04	0.05	1.27 (1.00 – 1.61)	1.48 (1.00 – 1.61)	2.02 (1.18 – 3.46)

ARI = Acute respiratory illness. SARI = Severe Acute Respiratory Illness. AFI = Acute febrile illness.

Among the group enrolled at PSCs, the overall hospitalization rate in the post-HBCT period was 2.5 times higher (RR 2.57, 95% CI 1.59 – 4.16) than the pre-HBCT period (Table 4). No changes were observed in the hospitalization rates of those HIV-infected not enrolled at PSCs or those who were HIV-uninfected.

## Discussion

Our study was unique in showing that a mass campaign of HBCT led to improvements in health-care seeking and decreases in incidence of some common infectious diseases among newly diagnosed HIV-infected persons. HIV counseling and testing is considered the entry point to HIV care, including access to cotrimoxazole prophylaxis and antiretroviral treatment [9,12,24-26]. For those persons who did access a PSC, the majority received appropriate treatment for HIV. The greater than 90% use of cotrimoxazole in those enrolled for HIV care in our study area was commendable and was in line with the Ministry of Health’s guidelines for universal use of cotrimoxazole irrespective of CD4 levels [27]. We found that 37% of newly-diagnosed HIV-infected persons who qualified for ARV (CD4 count <250 cells/mm^3^) had started this treatment.

Reductions in morbidity, mortality, HIV incidence and transmission, are the ultimate goal of large-scale efforts at counseling and testing as people may likely enroll for HIV care if they know they are HIV-infected [12,13]. Our study was one of the first to demonstrate a likely causal sequence between HBCT, accessing HIV care and treatment, and reduction in infectious disease morbidity. Among those newly diagnosed persons who accessed a PSC, we observed a 16% decline in incidences of diarrhea and acute febrile illnesses. The decline likely resulted, at least in part, from cotrimoxazole prophylaxis, which is known to reduce infectious disease morbidity [9,14,28-33]. Cotrimoxazole prophylaxis has been shown to reduce diarrhea in several previous studies in Africa, particularly from bacterial and parasitic causes [31-33]. Cotrimoxazole also has been demonstrated to be a potent prophylactic against clinical malaria, with reductions of more than 70% [31]. The Lwak area is a malaria holoendemic area, where both children and adults suffer from several clinical malaria episodes per year [19]. Cotrimoxazole also likely reduces the occurrence of other causes of AFI besides malaria, such as mycobacterial infections, bacteremia, and fevers of unknown etiology [32,33]. In addition to cotrimoxazole, initiation of ART has been shown to decrease both diarrheal illness and undiagnosed acute febrile illness in the first year after initiation of treatment. Additionally, ART leads to reduction in the incidence of known causes of AFI, such as bacteremia. We did not see a significant decrease in respiratory illness among those newly tested HIV-positive compared to the other two groups. This may be due to the fact that much of ARI is caused by viruses, which are generally not affected by cotrimoxazole prophylaxis. Severe ARI is a rarer syndrome and we might not have had the statistical power to detect a significant decrease in our sample. Moreover, there was a secular trend towards decreased SARI in the population, as witnessed in the HIV-negative group, which might have masked any specific impact of HIV treatment on SARI rates.

Care-seeking for illnesses increased more among the HIV-infected group in the post-HBCT period than the uninfected group. Care-seeking increased more at Lwak hospital than in the other three area clinics, which are run by the Ministry of Health (MOH). The greater increase in care-seeking at Lwak hospital might have been driven by several factors. First, the PBIDS field staff making the biweekly home visits encouraged study participants to go to Lwak hospital when ill. Second, Lwak hospital provides free high-quality care to enrolled study participants for all potentially infectious disease syndromes, including free hospitalization if needed, which is not available in other area MOH clinics [34]. Since access to free care was available at Lwak Hospital in pre-and post-HBCT periods, a key difference in motivation may, therefore, have resulted from an increased knowledge of one’s HIV positive status. Indeed, similar increases in attendance were not observed for the HIV-uninfected group who served as a comparison group to identify secular trends in health-care seeking in the pre versus post-HBCT period.

We believe that the observed increase in hospitalizations among those with HIV enrolled at PSCs was unlikely to be reflective of a true increase in severe disease among this group. As discussed, there was a general increase in healthcare seeking among HIV-infected persons, and therefore sicker patients were more likely to be hospitalized. This was particularly true at Lwak Hospital, because inpatient care was free for PBIDS participants and standardized clinical criteria were used for decisions on admission [16].

Approximately half of newly-diagnosed HIV-infected persons attended PSCs in the follow-up period. Many entered HIV care when their CD4 count was still relatively high (median 402 cells/mm^3^), which enhanced their ability to start prophylaxis before their disease had progressed. The reasons why half of those testing positive for the first time did not access HIV care are not clear. One possibility is that we missed some newly diagnosed persons who actually did attend a PSC. We only followed up persons in their homes one month after testing and then could only rely upon linking the names and residences of persons from HBCT with those in the registers of the area PSCs. This effort is limited by the fact that often persons use alternate names and change residences frequently [7]. It is also possible that individuals sought care in a clinic outside of the study area for reasons of anonymity. Second, there is likely still a barrier between HIV-testing and seeking care [7,35-37]. While doing HIV-testing in the home clearly overcame some of the obstacles of stigma, as well as the actual and opportunity costs of traveling to a fixed VCT center, the same obstacles would still exist for those seeking care at a PSC [6,37]. Further strategies are needed to improve the uptake of HIV care services after HBCT.

Our study had several limitations. Our data was limited to only one year of pre- and post-HBCT surveillance. It is possible that decreases in disease incidence among HIV-infected persons was due to annual variations in disease burden, which might not have been observed with more years of pre- and post-HBCT surveillance. However, the fact that we found differences in the reduction in disease between HIV-infected persons on treatment versus HIV-infected persons not on treatment and HIV-uninfected persons suggests that the reduction in disease was specific to receipt of HIV care and treatment. Second, the limited time periods of surveillance did not allow us to monitor the impact of care seeking on mortality as this is still a relatively rare event. Moreover, temporal factors might have influenced morbidity and mortality in this time period, particularly post-election violence that occurred in Kenya in 2008 [18]. Third, we assumed that the HIV status at the time of HBCT was the same for that individual in the year before and after testing. While we believe misclassification of HIV status was rare at the population level (1.5% annual incidence), it might have introduced slight bias in the morbidity findings due to acute illness symptoms with acute HIV seroconversion [38]. Lastly, we only followed-up HIV-positive clients at home one month after HBCT, which would not have documented those who sought care later; although we did search records for local PSCs later to try to capture those who might have registered later.

## Conclusions

In summary, we demonstrated that HBCT led to a substantial number (approximately 700 or 11% of first-time testers) of persons learning their HIV status for the first time. Newly diagnosed HIV-infected persons had a reduction in common infectious disease syndromes, likely through increased access to HIV care and treatment. While we did not demonstrate it, other studies suggest that HBCT, by improving access to HIV care and treatment, could lead to reductions in mortality and HIV transmission [12,24,25,30]. We believe such impact of HBCT on morbidity can be realized in other African populations in which many persons do not know their HIV status. However, we do caution that in order to realize the full benefits of HBCT enhanced efforts are needed to ensure those who test positive successfully enter HIV care and treatment.

## Abbreviations

AFI: Acute febrile illness; ARI: Acute respiratory illness; ART: Antiretroviral therapy; CDC: Centers for Disease Control and Prevention; HBCT: Home based HIV counseling and testing; HIV: Human immune-deficiency virus; KEMRI: Kenya Medical Research Institute; MOH: Ministry of Health; NASCOP: National AIDS/STD Control Program; PBIDS: Population-based infectious disease surveillance; PSC: Patient support centre; RR: Rate ratio; SARI: Severe acute respiratory illness.

## Competing interests

The authors declare that they have no competing interests.

## Authors’ contributions

GB, DF, RB, M Amolloh, M Ackers and BA participated in the conceptualization and design of the project, data collection, analysis and writing up of paper. GB, DF, KL, AA, DB, M Ackers participated in the review of the design of the study, development of study questionnaires, data collection, analysis and writing up of paper. GB, DF reviewed and wrote final manuscript. All authors read and approved the final manuscript.

## Pre-publication history

The pre-publication history for this paper can be accessed here:

http://www.biomedcentral.com/1471-2334/14/376/prepub
